# Supported online self-management versus care as usual for symptoms of fatigue, pain and urgency/incontinence in adults with inflammatory bowel disease (IBD-BOOST): study protocol for a randomised controlled trial

**DOI:** 10.1186/s13063-021-05466-4

**Published:** 2021-08-03

**Authors:** Christine Norton, Jonathan Syred, Sally Kerry, Micol Artom, Louise Sweeney, Ailsa Hart, Wladyslawa Czuber-Dochan, Stephanie J. C. Taylor, Borislava Mihaylova, Chris Roukas, Qasim Aziz, Laura Miller, Richard Pollok, Sonia Saxena, Imogen Stagg, Helen Terry, Zohra Zenasni, Lesley Dibley, Rona Moss-Morris

**Affiliations:** 1 King’s College London, 57 Waterloo Road, London, SE1 8WA UK; 2 Pragmatic Clinical Trials Unit, Queen Mary University of London, 58 Turner St, Whitechapel, London, E1 2AB UK; 3 NHS Digital, Skipton House, 80 London Road, London, SE1 6LH UK; 4 Health Psychology Section, Institute of Psychiatry, Psychology and Neuroscience, King’s College London, London, UK; 5 IBD Unit, St Mark’s Hospital, Watford Road, Harrow, HA13UJ UK; 6 Institute of Population Health Sciences, Queen Mary University of London, 58 Turner St, Whitechapel, London, E1 2AB UK; 7 Nuffield Department of Population Health, University of Oxford, Old Road Campus, Oxford, OX3 7LF UK; 8 Centre for Neuroscience, Surgery and Trauma, Blizard Institute, Wingate Institute of Neurogastroenterology, Barts and The London School of Medicine and Dentistry, Queen Mary University of London, 26 Ashfield Street, London, E1 2AJ UK; 9 Department of Gastroenterology, St George’s University Hospitals NHS Foundation Trust and St George’s University of London, London, SW17 0QT UK; 10 Department of Primary Care and Public Health Imperial College London, London, UK; 11 London North West University Hospitals, Watford Road, Harrow, HA1 3UJ UK; 12 Crohn’s & Colitis UK, 1 Bishops Square (Helios Court), Hatfield Business Park, Hatfield, Hertfordshire, AL10 9NE UK; 13 School of Health Sciences, Faculty of Education, Health and Human Sciences, University of Greenwich, (Avery Hill Campus), London, SE9 2UG UK; 14 Psychology Department, Institute of Psychiatry, Psychology and Neuroscience, King’s College London, London, UK

**Keywords:** RCT, Inflammatory bowel disease, Crohn’s disease, Ulcerative colitis, Fatigue, Pain, Faecal incontinence, Online self-management

## Abstract

**Background:**

Despite being in clinical remission, many people with inflammatory bowel disease (IBD) live with fatigue, chronic abdominal pain and bowel urgency or incontinence that limit their quality of life. We aim to test the effectiveness of an online self-management programme (BOOST), developed using cognitive behavioural principles and a theoretically informed logic model, and delivered with facilitator support.

**Primary research question:**

In people with IBD who report symptoms of fatigue, pain or urgency and express a desire for intervention, does a facilitator-supported tailored (to patient needs) online self-management programme for fatigue, pain and faecal urgency/incontinence improve IBD-related quality of life (measured using the UK-IBDQ) and global rating of symptom relief (0–10 scale) compared with care as usual?

**Methods:**

A pragmatic two-arm, parallel group randomised controlled trial (RCT), of a 12-session facilitator-supported online cognitive behavioural self-management programme versus care as usual to manage symptoms of fatigue, pain and faecal urgency/incontinence in IBD. Patients will be recruited through a previous large-scale survey of unselected people with inflammatory bowel disease. The UK Inflammatory Bowel Disease Questionnaire and global rating of symptom relief at 6 months are the co-primary outcomes, with multiple secondary outcomes measured also at 6 and 12 months post randomisation to assess maintenance. The RCT has an embedded pilot study, health economics evaluation and process evaluation.

We will randomise 680 patients, 340 in each group. Demographic characteristics and outcome measures will be presented for both study groups at baseline. The UK-IBDQ and global rating of symptom relief at 6 and 12 months post randomisation will be compared between the study groups.

**Discussion:**

The BOOST online self-management programme for people with IBD-related symptoms of fatigue, pain and urgency has been designed to be easily scalable and implemented. If it is shown to improve patients’ quality of life, this trial will enable clinicians and patients to make informed management decisions. This is the first trial, to our knowledge, focused on multiple symptoms prioritised by both people with IBD and health professionals.

**Trial registration:**

ISRCTN71618461. Registered on 9 September 2019.

**Supplementary Information:**

The online version contains supplementary material available at 10.1186/s13063-021-05466-4.

## Background

Inflammatory bowel disease (IBD) causes unpredictable bouts of gut inflammation, with acute illness, diarrhoea, and pain. In remission, many people with IBD live with fatigue, chronic abdominal pain and bowel urgency/incontinence [1]. There is no current cure for IBD, which usually starts in childhood or as a young adult. Most previous IBD research has focused on controlling inflammation. However, many people report continuing IBD-related fatigue (41%), abdominal pain (62%) and difficulty with continence (up to 75%) even when IBD is in remission [1–3]. Although patients in clinical remission may still have a burden of inflammation detected endoscopically or histologically, even patients with clinical and endoscopic/ histologic healing often continue to experience these symptoms. These symptoms limit peoples’ quality of life and ability to work and socialise. Patients feel that these symptoms are not taken seriously by health professionals and report that little help is given [2, 4, 5]. The James Lind Alliance IBD research priority-setting consensus put fatigue, pain and incontinence amongst the top 10 issues that IBD patients and clinicians want to be addressed by research [6].

## The IBD-BOOST programme of research

The current Randomised Controlled Trial (RCT) is stage 4 of IBD-BOOST, a UK National Institute for Health Research (NIHR) Programme Grant for Applied Research (PGfAR) funded programme (grant reference RP-PG-0216-20001). This programme aims to improve the quality of life of people with IBD by reducing the burden of IBD-related fatigue, abdominal pain and urgency/incontinence. Table 1 shows the four stages of the IBD-BOOST programme including this RCT.

**Table 1:** The IBD-BOOST programme of studies on fatigue, pain and urgency in IBD

**Stage 1** of the programme involved focus groups and interviews with people with IBD and IBD nurse specialists. In line with MRC guidance, these data were used alongside a theory-and person-based approach to develop a digital cognitive behavioural self-management intervention (IBD-BOOST). This stage is now completed.	
**Stage 2** of the programme involves a large cross-sectional survey of people with IBD to investigate the inter-relationships of IBD-related fatigue, pain and urgency/incontinence symptoms and the proportions wanting support to manage these symptoms. This stage is in progress and is not described further here.	
**Stage 3** of the programme is a non-randomised experimental study to test the effectiveness of a checklist and algorithm for identifying and treating medical causes of these IBD-related symptoms. The medical abnormalities detected in the study will be treated. This stage is not part of the current RCT and is not described further here.	
**Stage 4** (the current study) is an RCT of online self-management for symptoms of IBD fatigue, pain and urgency/incontinence (IBD-BOOST), with an embedded pilot study, health economics evaluation and process evaluation. Potential participants will already have completed the IBD survey (stage 2). Some of them will also have participated in stage 3.	

## Rationale for the choice of a cognitive behavioural intervention

Symptoms of fatigue, pain and urgency/incontinence have a major impact on quality of life of people with IBD [1–3]. Despite this, there has been remarkably little research on managing these troublesome IBD symptoms. Cognitive behavioural (CB) theories of these symptoms in other conditions suggest that disease factors trigger symptoms, but an interaction of cognitive, behavioural, emotional, environmental and physiological factors may strengthen and/or perpetuate them [7–10]. For instance, believing that pain and fatigue signal damage to the body (cognitions), may lead to avoidance of activity (behaviour) and distress (emotion). Distress activates the autonomic nervous system (physiology) which may generate additional symptoms, lead to poor sleep and perpetuate fatigue and pain. CB-based interventions which aim to alter these responses have been shown to reduce symptom severity and improve quality of life in other long-term conditions [11–13]. Online delivery of these interventions for chronic pain and fatigue appear effective in other conditions, with evidence of enhanced effects through the addition of minimal health care professional support [14–18].

Most studies in other medical conditions have focused on either fatigue or pain. Having separate CB interventions for each major symptom in IBD would create a substantive treatment burden. CB therapy (CBT) has been shown to be an effective treatment for irritable bowel syndrome (IBS) [12, 19]. As IBS is a multi-symptom condition, including abdominal pain alongside bowel disturbance, creating a single intervention to help manage all three of these IBD symptoms seemed justified. To test this empirically and in line the MRC framework for developing and testing complex interventions [20], we conducted a series of modelling studies and systematic reviews to define the specific cognitive and behavioural variables and treatment approaches that would benefit pain [21, 22], fatigue [2, 23] and urgency [21, 22] in IBD.

In separate studies, we have shown that severity and impact of fatigue and pain share several cognitive, emotional and behavioural correlates. For example, both pain and fatigue were found to correlate with depression, anxiety, focusing more on symptoms, interpreting these symptoms as signals of damage to the body and responding to symptoms with all-or-nothing or avoidance behaviours [22, 23]. All-or-nothing behaviour is when individuals exert themselves when symptoms are mild or absent, feel extreme fatigue or pain as a result and then avoid activity or rest up until symptoms subside again [23]. In IBD fatigue, psychosocial factors explained 41% of variance after controlling for disease status. In IBD pain, psychosocial factors explained 9.5% of variance in IBD pain severity and 24% of variance in IBD pain interference when controlling for demographic and clinical variables [22].

Similar processes have been observed in people experiencing bowel urgency, as hypersensitivity to rectal sensation leads to constant monitoring of rectal contents (hypervigilance) and anxiety in anticipation of incontinence [24]. Anxiety stimulates gut peristalsis and triggers a need to rush to the toilet; running makes incontinence more likely. Amongst people with IBD-related incontinence, 58% feel that anxiety worsens incontinence; even those who never experience incontinence worry about the possibility [3, 5]. The long-term impact of these psychological responses may perpetuate and maintain low mood and symptom chronicity, due to the physiological effects of the stress response, disrupted circadian rhythms, deconditioning and inflammation via the autonomic nervous system [10]. The aim of our intervention was therefore to target these transdiagnostic factors through evidence-based cognitive behavioural techniques focused on breaking vicious cycles, fostering self-management and improving quality of life [22].

Our modelling work also identified specific symptom-related factors which are important to include. For instance, to manage urgency in IBD, behavioural methods to strengthen the pelvic floor and bowel retraining techniques (such as practicing ‘holding on’) help to re-build confidence and the ability to defer defecation [25–27]. The intervention therefore needed to target both shared and symptom-specific cognitive and behavioural factors.

To provide an initial map for the content of the intervention, we drew the evidence base together in a conceptual logic model. We also drew content from previous evidence-based manuals for treating fatigue and pain in other conditions [10, 28–30] and a self-management booklet for incontinence in IBD [31]. Details of this process and the role of patient feedback to refine the intervention will be reported elsewhere (paper in development).

### Rationale for guided web-based self-management

People with IBD have been found to be willing to engage with, and complete, online self-management interventions [32]. In a 12-month trial in 333 patients, 79.6% completed the study and 88% finding this feasible and preferable to face-to-face care [33]. Some minimal health professional support has been shown to enhance engagement with self-management in IBD and other chronic conditions [34]. People with IBD want to take a greater role in self-management, including information that is both therapeutic and supportive [35]. Previous studies of online CBT in IBD have shown significantly improved quality of life, even when participants were recruited with no specific symptoms to be addressed [36].

In other chronic illnesses, self-management has been shown to improve management of cognitive symptoms and reduce fatigue, distress and social limitations [37]. CB models, theory and principles described above are ideally suited to self-management interventions, providing a clear structure to develop theoretical models and treatment mechanisms related to the outcome of interest (e.g. quality of life related interference of symptoms). CB models also map onto specific evidence-based techniques to enhance behavioural and cognitive change relevant to self-management [38, 39].

Our previous experience of recruiting people with IBD for interventions [31] and extensive patient engagement and literature on the popularity of electronic self-management in IBD determined our choice of online self-management. Although people want help with symptoms, they do not want repeated hospital visits and may decline help offered on that basis. We therefore designed BOOST as an online, tailored CB-based self-management programme which patients could complete in their own time at home with some minimal guided support from an IBD nurse trained in the intervention.

This protocol is reported in line with the SPIRIT (2013) statement. The SPIRIT Checklist can be found in Additional file 1.

## Methods: objectives, design and setting

### Objectives

The study aims to answer the following research questions:

- Primary research question:
  1. In people who report symptoms (scoring at least 5/10 on one or more symptoms on a 0–10 scale (where 0 = no problem and 10 = worst possible problem) and express a desire for intervention, does a facilitator-supported, tailored (to patient needs), online self-management programme for fatigue, pain and faecal urgency/incontinence in IBD improve IBD-related quality of life and global rating of symptom relief 6 months after randomisation, compared with care as usual?
- Secondary research questions:
  2. Is there any difference between the groups in severity of symptoms of fatigue, pain and urgency/incontinence at 6 and 12 months after randomisation?
  3. Is there any difference between the groups in IBD-related quality of life and global rating of symptom relief 12 months after randomisation?
  4. Does prior medical optimisation of symptoms (in stage 3 of this programme) moderate the treatment response (i.e. do those receiving medical optimisation show greater treatment gains than those who do not) as measured by the primary outcomes?
  5. Do people with IBD in remission at trial commencement have a better response to treatment (primary outcomes) than those with active disease? (remission defined as faecal calprotectin within normal range (200 or under) and /or IBD control score [40] of 13 or over.
  6. Do baseline depression, or the presence of irritable bowel syndrome (Rome IV criteria) moderate treatment response to intervention (primary outcome measures)?
  7. Do changes in illness perceptions and behaviours, IBD specific anxiety and self-efficacy, and depression from baseline to 6 months mediate intervention effects on the primary outcomes at 12 months?
  8. Is a facilitator-supported, tailored, online self-management programme for fatigue, pain and faecal urgency/incontinence in IBD cost-effective?
  9. What are patients’ expectations and experiences of the intervention and what factors may have influenced the intervention implementation (qualitative work in process evaluation)?

### Hypothesis for the RCT

A facilitator-supported, online, tailored self-management programme for fatigue, pain and faecal urgency/incontinence in people with IBD will result in better quality of life compared to care as usual at 6 months after randomisation.

### Design for the main RCT

**Fig. 1:**
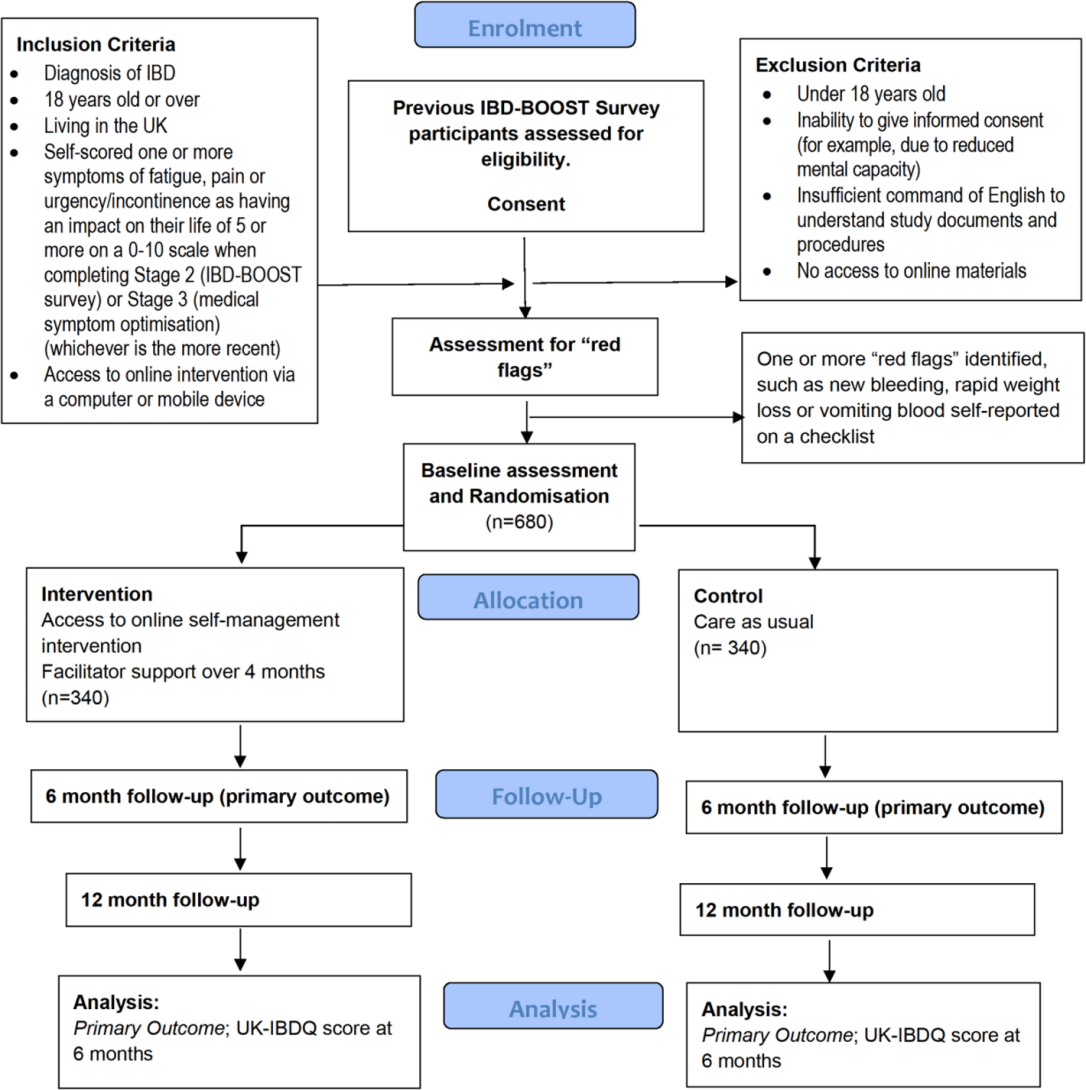
Consort diagram for a randomised control trial of supported self-management for symptoms of fatigue, pain and urgency/incontinence in people with inflammatory bowel disease

The design is a pragmatic multi-centre two-arm, parallel group superiority RCT. We will compare facilitator-supported online self-management versus care as usual (CAU) to manage symptoms of fatigue, pain and faecal urgency/incontinence in IBD. There are one baseline assessment and two assessments at 6 and 12 months after randomisation. Primary outcomes are as follows: IBD quality of life and global rating of symptom relief at 6 months. The CONSORT diagram for the RCT is given in Fig. 1.

### Internal pilot study

An internal pilot study will test planned methods and procedures for the main RCT and identify barriers to the components working together and potential recruitment issues. We will recruit and randomise the first 100 participants in the RCT, aiming to complete this within the first 6 months of the RCT recruitment. The methods planned for the main RCT will be followed. If no substantive changes are made, these 100 participants will be included in the main RCT analysis. Note: this pilot has been a little delayed by the Covid-19 pandemic and will now cover the first 10 months of the RCT.

### Study setting

The study will be conducted at National Health Service (NHS) hospital sites in England with IBD services and at King’s College London (KCL). A full list of sites is available from the Chief Investigator on request.

## Methods: participants, interventions and outcomes

### Identifying participants

People with IBD with symptoms of fatigue, pain and/or urgency will be recruited from people responding to our earlier IBD-BOOST survey (stage 2 of the programme) or from people who have completed the survey plus medical symptom optimisation study (stage 3 of the programme), who request further intervention for these symptoms and who meet our eligibility criteria (below). The survey was sent by post or an electronic link in an email to unselected patients with IBD by IBD clinic hospital sites, the UK national IBD BioResource or the charity Crohn’s & Colitis UK. The completed surveys were returned by post or electronically.

The central IBD-BOOST research team will screen the IBD Survey (stage 2) responses and the follow-up questionnaires of participants in the IBD-BOOST Optimise study (stage 3) who have consented to be contacted for further research. Sufficient English and capacity for consent will be presumed from the previous response to the survey. A link to the online consent form for those who wish to participate in the RCT will be embedded in an email from a member of the central research team. If the online database is not available, paper copies of the Participant Information Leaflet and consent form will be sent.

### Eligibility criteria

#### Inclusion criteria

- Diagnosis of IBD (self-reported as having been medically diagnosed with IBD including patients with an ileo-anal pouch or stoma)
- 18 years old or over
- Living in England, Scotland or Wales
- Have participated in stage 2 of the programme (IBD-BOOST survey) and have self-scored one or more symptoms of fatigue, pain or urgency/incontinence as having an impact on their quality of life of 5 or more on a 0–10 scale when completing stage 2 (IBD-BOOST survey) or stage 3 (medical symptom optimisation) (whichever is the more recent)
- No ‘red flags’—see below
- Access to the online intervention via a computer or mobile device

#### Exclusion criteria

- One or more ‘red flags’ identified on pre-randomisation screening (such as new bleeding, rapid weight loss or vomiting that has not been previously reported to a health care practitioner), self-reported on a screening checklist. If ineligible because of a red flag, a participant may be re-assessed if they contact the research team and report that the information they originally provided has changed such as the symptom has been adequately investigated or managed, in which case the participant can be included.
- Inability to give informed consent (for example, due to reduced mental capacity)
- Insufficient command of English to understand study documents and procedures

### Screening for ‘red flags’ and other risks

After participants consent, they will self-complete an online or paper copy checklist to check eligibility criteria above. Following consent, we will screen patients for ‘red flags’. These are potentially serious issues identified by our clinical co-investigators that may be undetected underlying causes of symptoms. If a participant’s responses indicate a potential red flag, the participant will be advised to seek a consultation with their health care team and only recruited to the trial once the symptoms have been adequately investigated.

We will also send consenting participants who have not already taken part in IBD-BOOST Optimise (stage 3 of the programme, who will have already done this) a kit for testing the faecal calprotectin level (see below and Additional file 5), but non-response to this test will NOT delay randomisation.

Additionally, item 9 (suicidal ideation) of the PHQ-9 in the baseline questionnaire (see below) will be reviewed to assess for risk of harm/suicidal ideation. If the participant has scored 2 or 3 (indicating frequent thoughts around suicidal ideation) then a risk assessment will be carried out by a psychologist by telephone. Participants assessed as low or medium risk will be able to continue into the trial, reminded of resources to access for further support and provided with an optional template letter to notify their GP. Appropriate urgent action will be taken for patients assessed as high risk and these individuals will not proceed into the trial.

### Study interventions

BOOST is a web-based programme for self-management of pain, fatigue and urgency/incontinence symptoms in IBD based on the principles of CBT. ‘BOOST’ was initially mapped out based on the theoretical models described above and then developed with extensive patient and IBD specialist nurse input. BOOST has undergone extensive user feasibility testing prior to the pilot study, as recommended by the Medical Research Council when developing complex interventions [20].

BOOST includes 12 online sessions which can be viewed on computer, smart phone or tablet (however, participants are advised that computer or tablet is likely to give a better view of the programme). The content has been developed to be interactive and tailored to patients’ needs. A number of logic pathways have been programmed into the programme alongside self-assessments, so the computer only provides content and tasks relevant to issues identified by the participant.

Sessions 1–7 are core sessions to be completed by all participants experiencing fatigue, pain and faecal urgency/incontinence. Based on a Cognitive Behavioural model of IBD symptoms, the core sessions cover topics around understanding IBD symptoms, balancing activity and exercise, sleep hygiene, changing negative thoughts, coping with stress and emotions, and making the most of social support. These core sessions have associated tasks for participants to complete in between sessions in their day-to day-life, such as working toward completing goals or keeping a sleep diary. Sessions 8–11 are symptom-specific sessions to be completed by participants experiencing or with a specific interest in fatigue, pain and faecal urgency/incontinence, respectively. Some of the content of the website was adapted from previous manuals using a similar approach for managing specific symptoms in IBD or with different illness populations [31, 41–46]. Table 2 provides a summary of each session for all BOOST sessions. The symptom-specific sessions provide participants with more in-depth psychoeducation on the interaction between medical and psychosocial factors contributing to the severity and impact of the specific symptom, together with practical tips and ongoing exercises on how to better manage them.

**Table 2:** Intervention sessions

**Session 1.** Understanding your IBD symptoms	Factors that can contribute to fatigue, pain and urgency in IBD. Identifying specific factors and developing a personal vicious cycle of symptoms. Use of self-monitoring not symptom focusing. Setting aims for the programme. *Task: Symptom severity and stress level diary.*
**Session 2.** Balancing your activity, eating and exercise	Importance of activity and exercise. How fear leads to avoidance. Eating patterns. Setting goals for activity and exercise. *Task: Reviewing and working toward goals for activity + sleep diary.*
**Session 3.** Improving your sleep	Why is sleep important? Sleep patterns & habits. Improving sleep. Setting goals for sleep. *Task: Reviewing and working toward goals for sleeping patterns and habits.*
**Session 4a.** Changing your thoughts: Part 1	Why are thoughts important? Identifying unhelpful thinking. *Task: Thought record.*
**Session 4b**. Changing your thoughts: Part 2	Developing alternative thoughts. *Task: Alternative thought record.*
**Session 5.** Managing stress and coping with emotions	The effects of stress and finding ways to manage it. The role of emotions and determining how best to take care of oneself. Setting goals for managing stress and emotions. *Task: Reviewing and working toward goals for stress management + stress diary.*
**Session 6.** Making the most of your social support and communication	Types of social support. Communication and disclosure. Setting goals for social support. *Task: Reviewing and working toward goals for social support.*
**Session 7.** Managing and understanding fatigue in IBD	Types of fatigue. Factors related to IBD fatigue. Exploring a vicious cycle of IBD fatigue; the role of thoughts, emotions and behaviours.
**Session 8.** Managing and understanding pain in IBD	Difference between acute and chronic pain in IBD. Factors related to IBD pain. Exploring a vicious cycle of IBD pain; the role of thoughts, emotions and behaviours. Common questions around pain in IBD.
**Session 9.** Managing urgency and leakage	Bowel functioning and bowel control difficulties. Stress and anxiety in urgency. Exercises to help reduce accidents. Practical bowel management tips. Using social networks to help manage urgency.
**Session 10.** The role of acceptance and self-compassion in pain	What is acceptance and how can it help? The role of resilience. Practical exercises.
**Session 11.** Summary and maintaining improvement	Reviewing programme aims. Preparing for the future. Sustaining and building upon improvements.

Participants will be able to log into BOOST via a computer, tablet or smart phone, according to their preference, and advised to complete approximately one session per week. Each session takes 30–60 min to complete. Participants will be able to complete the sessions at a time and place that is convenient to them and to pace themselves in a way that suits them and their lifestyle.

#### Facilitator support

During the intervention, the participants will be supported by an assigned healthcare professional, who will act as their facilitator. Facilitators will be recruited (and trained) from our 15–20 sites, aiming for at least 2 per site, enhancing generalisability. Facilitators will predominantly include IBD nurses and a small number of academic psychologists within the research team in instances where nurses are not available, for instance supporting participants recruited through non-hospital sites.

The programme suggests one 30-min phone call after participants have completed session 1, and then online messaging from the participant to the facilitator as the participant wishes thereafter. During the phone call, the facilitator will review the participant’s personal vicious cycle completed in session 1, guide the participant’s understanding of factors contributing to symptoms from a cognitive behavioural model and review the participant’s programme aims. Each facilitator will use a telephone checklist prompt sheet to guide the telephone call. At the end of each online session, the participant is prompted to consider if they want to send a message. The facilitator can reply to online messaging within the intervention platform. The facilitator will have access to information on how participants are using the intervention (sessions and task completed) and will monitor and help to promote participants’ engagement with the intervention, together with supporting participants work toward achievement of their intervention goals. Facilitators will be encouraged to log in weekly to check their participant’s completion of sessions/tasks and send any necessary messages to promote engagement, as well as reply to messages received.

- *Group 1 (intervention)*: Access to care as usual plus the online tailored BOOST self-management programme for 6 months, plus one individual telephone or Skype support session (for up to 30 min, training and a paper copy of the content will be provided for the facilitator), plus access to online messaging with their facilitator via the BOOST platform for the first 3 months after recruitment.

Participants will log in to the online programme (unique login), undertake symptom self-assessment, prioritise what symptom they want to work on, with signposting to relevant sections depending on symptoms and response to online questions. (Example: link to a section on improving sleep patterns for those with fatigue who nap a lot in the day or with a problem getting to sleep at night).

- *Group 2 (control)*: Care as usual (CAU).

All participants will have access to all usual IBD care, including monitoring with clinic visits and/or via the local IBD helpline (this will constitute care as usual for group 2).

Participants in group 1 with an active IBD flare during intervention will not be expected to discontinue the intervention and will have continued access to the online programme. All randomised participants, regardless of whether they have reported a disease flare or not, will be sent the questionnaires to capture outcome measures at 6 and 12 months after randomisation.

At 12 months, participants allocated to CAU will be offered access to the online intervention (but no facilitator support). Uptake within 6 months will be reported.

For use of primary and secondary healthcare, other health care resources and medication use will be collected using the baseline, 6- and 12-month follow-up questionnaires (see Economic Evaluation section below).

We have worked with our patient panel to devise strategies to optimise engagement with the online intervention and retention of our sample, such as personalised text or email reminders.

#### Facilitator training and intervention fidelity

There will be specific training for facilitators on delivering the intervention and the principles of CBT via a manual (which contains the full text of the online intervention for reference) and a 1-day intensive course delivered by expert psychologists in the intervention development team. The training will introduce facilitators to the principles of a cognitive behavioural approach, provide opportunity to observe and practice telephone call role plays and review example models of vicious cycles of symptoms which participants will construct during session 1. The training will also cover responding to online messaging, and what to do in certain situations (e.g. the participant not engaging with the programme, getting stuck or frequent or persistent use of the messaging service). Adverse events will be discussed and the manual contains standard operating procedures (SOPs) for actions to be taken in certain scenarios, such as the participant appearing very depressed or suicidal.

Facilitators will receive further training and supervision during the site initiation visit from the intervention development/trial team. Prior to starting the trial, facilitators will also receive one-to-one supervision on a ‘practice patient’ (a person with IBD from our Patient and Public Involvement panel) over a 2-week period. A practice telephone session between facilitator and patient will be audio-recorded for training purposes, where facilitators will use the telephone checklist prompt sheet to guide the call. Facilitators will receive feedback from a member of the intervention development team on the telephone conversation and online messaging responses with the practice patient.

Online messaging contacts will be recorded and analysed in the process evaluation (below). Facilitators will receive monthly supervision (or on request of the facilitator) with a trained member of the research team to ensure fidelity and provide support for any difficult issues that may arise. Supervision will include reviewing online messaging and tasks. Participants are told when they commence the online programme that their facilitator and the supervisor will be able to see their messages and tasks. Facilitators will complete the telephone checklist prompt sheet for each participant and provide these to the research team, and receive supervision on a trial patient telephone call, upon the patient’s permission for a psychologist to listen (but not audio record). At the end of the trial, a randomly selected sample of online messages will be rated for fidelity by an independent rater (see process evaluation below).

#### Discontinuation criteria

We do not have any criteria for discontinuing a participant.

### Outcomes

Trial outcome measures have been selected following focus group opinions and refined with our patient panel.

### Primary outcome measures

UK Inflammatory Bowel Disease Questionnaire (UK-IBDQ) [47] and global rating of symptom relief at 6 months after randomisation.

### Other measures (at baseline, and as secondary outcomes at 6 and 12 months after randomisation)

- UK-IBDQ [47] at 12 months
- Rating of satisfaction with results of IBD-BOOST programme (simple 0–100 visual analogue scale) at 6 and 12 months only
- Global rating of symptom relief at 12 months
- Numerical (0–10) pain rating scale
- Vaizey (faecal) incontinence score [48], reflecting patients’ perceptions of severity [49]
- IBD-Fatigue score [50, 51]
- IBD-Control score; 8-item self-reported score to measure disease control from the patient’s perspective [40]
- EQ-5D-5L [52] general health-related quality of life at baseline and 6 and 12 months after randomisation

### Health economic measure (at baseline, and 6 and 12 months after randomisation)

- IBD Resource Use Questionnaire to determine health care and other resource use and costs due to IBD, including visits or phone calls to primary and secondary healthcare services, medication use and personal expenses.

### Moderators and mediators

These include measures which may either moderate or mediate the treatment response. These data are important in terms of variables which may help to explain response, to be used as part of process evaluation (see below): (at baseline, 6 and 12 months after randomisation):

- Symptoms which determine a co-diagnosis of irritable bowel syndrome (IBS) and IBD: Rome IV criteria for IBS
- Medications for depression, pain and diarrhoea
- Measures of change in general mood: depression as measured by the Patient Health Questionnaire (PHQ-9) [53]. This measure includes a question (item 9) that may indicate suicidal thoughts. The action to be taken if suicidal thoughts are indicated is described in ‘Assessment and management of risk’ below.
- To measure self-efficacy: Self-efficacy for managing chronic disease 6-item scale [54]
- To measure IBD/illness specific cognitions: Brief Illness Perceptions Questionnaire (BIPQ) [55] (minus the open ended causal items). Items are prefaced in relation to beliefs about IBD symptoms rather than IBD generally.
- Medications for depression, pain, diarrhoea
- To measure IBD/illness specific affect: Visceral Sensitivity Index (VSI): gastrointestinal symptom-specific anxiety [56]
- To measure behaviour: All-or-nothing and avoidance/resting subscales from the Cognitive and Behavioural Responses to Symptoms Questionnaire (CBRQ)

Note: these outcome sets are questionnaires validated for self-completion and will be compiled into a single online questionnaire (available from the Chief Investigator). We recognise the potential burden on participants of these multiple outcome measures (just under 150 questions). However, as we are assessing multiple symptoms and are keen to include potentially explanatory variables, we feel that these are necessary. We have worked with our PPI representatives on optimising format for outcome measures in a user-friendly online interface.

#### Participant timeline

This is shown in Table 3. There are no face-to-face visits; all study procedures are completed remotely by post, telephone or online messages.

**Table 3:** IBD-BOOST RCT screening and data collection schedule (SPIRIT figure)

	*Pre-consent*	*Pre-baseline*	*Baseline*	*6 months*	*12 months*	*Ongoing or during treatment*
Screen for eligibility	X					
Study within a trial (SWAT) randomisation	X					
Invite reply	X					
Consent form		X				
Red flags assessment		X				
Adverse event form						X
Drop-out event form						X
Faecal calprotectin test		X				
Baseline case report form			X			
Follow-up case report form				X	X	
Participant qualitative interviews			X		X	
Clinician qualitative interviews			X		X	
Randomisation			X			
Nurse support				X		
Intervention website user query				X		

#### Sample size

Our primary outcome is the UK-IBDQ, range 0 to 120 where low values indicate poor quality of life. Using several published studies [47, 57, 58], we estimate the standard deviation of the change in score to be between 20 and 30. This would mean an effect size of 0.3 would equate to a difference of between 6 and 9 points on the scale. In the validation study [47], the difference in score between those with mild disease and disease in remission was 12 points and the effect of relapse was 10 points.

An effect size of 0.3 was observed in a small study of dietary advice in patients with ulcerative colitis [57].

We will randomise 680 patients, 340 in each group. Assuming a 20% loss to follow-up, there will be 270 in each group in the analysis. Assuming there are 30 facilitators, they will each support ~ 11 patients on average of whom ~ 9 will contribute to the analysis. If the ICC for clustering between facilitator is 0.04, the design effect will be 1 + 0.04 × 8 = 1.32 and the effective sample size in the intervention arm is 270/1.32 = 204. 270 in the control and 204 in the intervention arm would allow an effect size of 0.3 to be detected with 90% power.

A 20% drop-out assumption is based on drop-out rates from previous studies of self-management: 19.2% of 682 participants in IBD disease self-management (not online) [59]; 20.4% of 333 participants for online self-management of ulcerative colitis disease flares [33]; 18% control and 16% intervention of 1140 participants randomised for chronic disease self-management in other diseases [37].

#### Recruitment

The previous stage of our programme involves a large-scale survey about symptoms to people with IBD. We will continue sending out batches of this survey until we achieve adequate recruitment for the RCT. Respondents to this separate survey who are apparently eligible to participate in the RCT will be sent an invitation, a participant information sheet, a consent form and a further pre-randomisation eligibility checklist for ‘red flags’ (see eligibility criteria above) electronically or by post. Once a patient has consented and is eligible, a baseline questionnaire is sent.

## Methods: assignment of interventions

### Randomisation procedures

Participants who consent, are eligible and return the baseline questionnaire (which is different form the survey they will have already completed) will be randomised by the central research team using an online randomisation system developed for the study by the PCTU. Allocation will, therefore, be concealed until consent and baseline measurements have been completed. It is not possible to blind participants or facilitators once randomisation has occurred, due to the nature of the intervention.

The central team will inform the participant which group they are in and inform the clinical sites of participants in group 1 who will receive local facilitator support (phone call and online messaging). The local facilitator will be given the participant’s details and access to their online tasks.

Stratified central web-based randomisation to 2 groups. Stratified by:

- Diagnosis (Crohn’s disease vs. any other type of IBD, including ulcerative colitis and IBD-unclassified)
- Whether or not participated in the stage 3 study in this programme (medical symptom optimisation)

Random block sizes of 4 and 6 will be used. Block sizes will not be disclosed to ensure allocation concealment.

### Blinding procedures

Faecal calprotectin level will be entered into the database by a person blinded to group allocation when the result has been returned from the laboratory and the participant informed of the result. Blinding of participants or facilitators is impossible. Data cleaning for the outcome measures will be by a statistician blinded to group allocation. Blinding beyond this will be impossible as data is clustered by the facilitator in the intervention arm only.

## Methods: data collection, management and analysis

### Data collection methods

Outcome measures will be collected by either direct participant input into a secure online study-developed database managed by the QMUL Pragmatic Clinical Trials Unit (PCTU) or via paper copies sent by central research team at KCL. Paper copies received in the post will be inputted manually into the database by the research team at KCL. The questionnaires will not be available in any languages other than English. Each clinic or site will complete a study log for all their participants who have been contacted for the study. Two email or text reminders will be sent to non-responders. If there is still no response, an attempt will be made to collect the primary outcome measure by telephone.

Window for return of outcome measures is as follows: up to 8 weeks (with reminders) at the 6-month follow-up and up to 4 weeks (with reminders) at the 12-month follow-up (with up to 2 text or email reminders). If primary outcome measure is not returned by 8 weeks after the 6-month time point, an attempt will be made to have this (the IBDQ and global rating of symptom relief only) completed over the phone by a member of the central trial team reading out the questions and recording the responses.

Baseline demographic details (age, gender, type of IBD) and ROME IV criteria for irritable bowel syndrome [60] will be collected.

We will adopt evidence-based methods to minimise loss to follow-up which have been identified in a systematic review [61]. These include providing incentives to participants, contacting respondents prior to sending the follow-up questionnaires and using phone, text and email to contact participants. To optimise our response rates, we will provide an unconditional inconvenience payment of £5 at 6 months and 12 months by post with a letter at the same time as we send out the follow-up questionnaire electronically or by post.

### Data management

Data will be stored on a secure database. Participants will complete the outcome measures via an online link and will input their data directly into the database or complete paper copies and send via a provided stamped addressed envelope. Further details are available from the Chief Investigator. All interviews will be digitally recorded, anonymised, professionally transcribed verbatim and analysed using, if appropriate, NVivo8 software for data management.

### Statistical methods

#### Methods of analysis

Baseline demographics, type of IBD classification, Rome IV IBS criteria and baseline values of outcome variables will be presented for both study arms using descriptive statistics only.

The UK-IBDQ and global rating of symptom relief will be compared between the two study arms at 6 and 12 months after randomisation (analysis conducted after 12-month data collection point). The following covariates will be included in the model: baseline value of outcome measures, stratification factors, fatigue, pain and incontinence at baseline, age and gender. Facilitators will be added as a random effect in the intervention arm only.

Secondary outcomes will be analysed in the same way with inclusion of baseline value of respective outcome. Outcomes and covariates may change in the light of new information but will be agreed prior to unblinding of the data.

Pre-specified subgroup analyses to investigate subgroups who might respond better to treatment will be conducted as part of the process evaluation (see below).

A sensitivity analysis using imputation methods to allow for missing data and reasonable assumptions for those lost to follow-up will be carried out.

A detailed statistical analysis plan will be completed and signed off by the Programme Steering Group prior to unblinding of data.

## Economic evaluation methods

An economic evaluation using recommended methods [62] will be undertaken from the National Health Service /Personal Social Services and patients’ perspectives to evaluate the cost-effectiveness of the intervention at 12 months. The cost of the intervention, including facilitator’s contacts with each patient, website hosting and maintenance and facilitators’ training (venue, trainers’ and facilitators’ time, travel and subsistence, administrative support and materials) and supervision, will be evaluated.

Data on the use of health, social care and other services will be collected from all study participants using a study-specific IBD Resource Use questionnaire, developed within this Programme Grant, asking about contacts with primary and secondary care, investigations, medications, hospitalisations, employment and out-of-pocket expenses.

Unit costs will be sourced from national sources (e.g. Personal Social Services Research Unit: Unit Costs of Health and Social Care; Department of Health: NHS reference costs; British National Formulary; NHS Improvement: National tariff payment system) and will be applied to categories of resources used to estimate individual participant total costs. Health-related quality of life data will be collected using the EuroQoL EQ-5D-5L questionnaire.

We will follow the intention-to-treat principle, and missing data will be handled using multiple imputation approach. We will calculate quality-adjusted life years (QALYs) and total costs for each participant in the trial during the 12 months follow-up. We will evaluate the incremental QALYs and costs with allocation to the BOOST intervention and the intervention’s cost-effectiveness at 12 months follow-up. The costs and EQ-5D-5L utilities will be compared between the two study arms at 6 and 12 months after randomisation, with adjustments made for baseline values of outcome measures (costs in previous 3 months and EQ-5D-5L at baseline), stratification factors, fatigue, pain and urgency/incontinence at baseline, age and gender. Facilitators will be added as a random effect in the intervention arm only.

An incremental cost-effectiveness ratio (ICER) will be estimated as the additional cost per an additional QALY gained. Uncertainty around the ICER point estimate will be assessed [63]. The probability of the online IBD self-management intervention being cost-effective compared to usual care will be estimated at the NICE threshold values of £20,000 to £30,000 per QALY gained. One-way sensitivity analyses will be conducted to explore the uncertainty associated with the cost of the intervention and use of healthcare services.

A health economics analysis plan, specifying the health economics analyses in detail, will be finalised and signed off prior to unblinding of data.

## Methods: process evaluation

### Research question

What factors (promoters and blockers, individual and organisational) impact on the completion and effect of the intervention?

A mixed methods process evaluation of the RCT will be conducted based on the MRC guidance [64]. The design of the process evaluation is based upon the logic model and consequent discussion within the study team, so that the process evaluation can explore the areas of greatest uncertainty.

### Aims

To investigate the processes through which the intervention is delivered, and what is actually delivered in practice, to aid the interpretation of the results of the main trial and to inform future rollout and implementation of the intervention, if successful.

### Objectives of the process evaluation

- Determine expectations at baseline through interviews
- Determine if the intervention was delivered as planned (intervention fidelity)
- Explore participant responses to the intervention both quantitatively (through routinely collected study process data) and qualitatively (through interviews)
- Explore participants’ responses to being in the control arm of the study through interviews
- Understand IBD nurses’ views of the intervention and its integration and usability in everyday NHS care through interviews
- Consider any potential contextual influences on the intervention implementation and outcomes.

Based on the logic model developed for the intervention, which provides a detailed description of the intervention and its causal assumptions, we will seek to monitor intervention fidelity and provide insights into how the intervention did or did not work in practice, any unintended consequences, as well as providing information to aid future implementation and dissemination. A list of key assumptions and uncertainties has been developed by the intervention development team. These will be explored and tested in this process evaluation.

The process evaluation will run concurrently with the RCT in a largely ‘passive’ model [64]. However, this passive model will not be operational during our internal pilot study and the teams will work closely together during the pilot to address any issues raised.

### Adherence

#### Fidelity to the protocol

Proportion of people randomised to the intervention who clicked on the link to the online intervention and commenced the intervention and completed a minimum of four online sessions will be considered to have adhered to the intervention. We will also assess the date the baseline CRF is completed, date ‘red flags’ were completed and the date randomised to check study processes worked as intended.

### Quantitative

- Monitoring online log-ins (number and spacing/timing of log-ins and time spent on each section and in total): automatically collected by the programme software, enabling us to see how participants interact with the intervention.
- A record of number and time of facilitator telephone support sessions or emails.
- Evaluation of moderators and mediators of treatment effect section, below gives details of quantitative analysis of possible moderators and mediators of response to intervention.

### Qualitative

- Fidelity to the intended facilitator support: a sub-set of facilitators’ messages will be analysed and compared with instructions in the training manual for fidelity to the intended facilitator support. All facilitator interactions online with participants will be captured and randomly selected interactions (messaging) will be stripped of all personal identifying data and subjected to content analysis [65], to enable assessment of intervention fidelity (as defined by the training day and intervention manual).
- Face-to-face, telephone or Skype interviews with a purposive sample of up to 30 recruits (or until apparent data saturation) before and after the intervention (attempting to recruit the same people before they know their group allocation and then the same people 6 months later; if it is not possible to recruit people for a second interview at 6 months (or when they drop out if this is sooner), these participants will be replaced by another participant who was allocated to the same group with broadly the same characteristics). Interviews will be completed at different times after recruitment, to understand expectations and experiences of the intervention, its acceptability and which aspects they felt were most or least helpful (informing adjustment to the intervention before future rollout), and for their opinions on changes in their cognitions and behaviours. We will also interview at least two thirds of the facilitators who are IBD nurse specialists (approx. 20: or until apparent data saturation) to understand their views on supporting the online intervention and fit with their existing workload.

Participants who have indicated on the trial consent form that they are happy to be approached about interviews will be purposively selected and consented for interviews. Facilitators indicating willingness will be interviewed. The topic guides for interviews have been developed with our PPI panel. Data will be analysed iteratively and as the interviews progress the topic guide will be adapted, based on themes which emerge from earlier interviews, to enable exploration of issues which appear relevant in later interviews.

The interview sample will be purposively selected to include both sexes, a range of ages, both IBD diagnoses and those who completed or did not complete (the intervention), or were in the CAU group to better understand patient perspectives on the intervention, experiences of being in a wait list control and whether this altered behaviours and cognitions around symptoms, whether non-completion was due to the design and demands of the intervention, or due to other factors.as well as any unanticipated pathways and consequences. For analysis, interpretive data analysis will be informed by the Analytical Hierarchy Framework (AHF) [65], guiding methods for handling, analysing and generating findings from qualitative data.

We will train patient volunteers to co-analyse anonymised qualitative data (as we have done successfully in other studies [4, 66–69]), and help us interpret and present our findings widely to patient and media audiences. NVivo software will be used to manage data and enable sorting, labelling and retrieval of data segments prior to the human endeavour of interpretation and representation of findings.

The process evaluation team will work to integrate these sources of qualitative and quantitative data into a coherent report which seeks to illuminate the results of the RCT. There will also be a mediation analysis (see above).

### Evaluation of moderators and mediators of treatment effects

The purpose of this additional quantitative process analysis is twofold:

1. To explore moderators of intervention effect

This will allow us to assess if there are any key subgroup effects and if the intervention should in future target specific groups of patients. For instance, if depression and disease activity moderate treatment effects, it may be best to focus the intervention on patients in remission and provide alternate treatment to people with depression. If medical treatment optimisation moderates outcome, then again this confirms best practice would be to provide optimised medical treatment before the intervention. Alternately, if there are no significant moderators of effects, the intervention may be generalisable to the broader IBD population.

Subgroup analyses to be investigated are those who underwent Optimise (stage 3); IBD in remission (see definition above); baseline measure of anxiety and depression (PHQ-9 and VSI); and ROME IV criteria for IBD met at baseline or not. These will be investigated by adding interactions terms to the analysis models used for the primary outcomes (UK- IBDQ and global symptom relief).

2. To explore mediators of change

Mediation analysis using structural equation models allows us to explore if a treatment effect occurs through hypothesised treatment mechanisms. We have based our choice of mediator measures on our cognitive behavioural model underpinning the intervention and described in our logic model (available on request form the lead author). Putative mediators have been mapped onto the key intervention components, i.e. the factors the intervention attempts to target to bring about improvements in symptom and quality of life. These include the following: the Visceral Sensitivity Index (VSI [56] and PHQ-9 [53] for symptoms of anxiety and depression; Self-efficacy for managing chronic disease 6-item scale [54]; Brief Illness Perception questionnaire [55]; All-or-nothing and avoidance/resting subscales from the Cognitive and Behavioural Responses to Symptoms questionnaire (CBRQ); IBD-Control [40]; faecal calprotectin [70]; satisfaction with outcome.

We are looking at whether proximal change in the mediators at 6 months predicts improvement in the outcomes at 12 months.

## Methods: monitoring

### Data monitoring

A study monitoring and auditing plan will be produced. The risk assessment for the trial will be under continual review to assess relevance and applicability and to identify any actions that may be required. The study may be subject to inspection and audit by the sponsor and other regulatory bodies to ensure protocol compliance and adherence to GCP and the UK Policy Framework for Health & Social Care Research 2017. Protocol deviations, non-compliances or breaches from the approved protocol must be reported to the Sponsor R&D Office and PCTU within 24 h of becoming aware of the event.

### Harms

#### Assessment and management of risk

This is a low-risk study (as assessed by PCTU), although there is potential for participants to become distressed when thinking about their symptoms. The intervention site will include a link / website address to CCUK who provide support via their helpline, and contact details are included in the Participant Information Leaflet. The outcome measures include questions on anxiety and depression and the PHQ-9. The central research team will monitor responses to suicidal thoughts and contact the patient and undertake a risk assessment if necessary. At the end of the questionnaire, there are helplines listed that can offer support.

## Risks/benefits

### Stopping the trial

There is no Data Monitoring Committee for this low-risk study. The trial may be prematurely discontinued by the Sponsor or Chief Investigator on the basis of new safety information or for other reasons given by the Programme Steering Committee or REC concerned. There will be no formal stopping rules based on the intervention outcomes. In the unlikely event that the study is prematurely discontinued, active participants will be informed and no further participant data will be collected.

#### Adverse events (AEs)

Adverse events will be assessed using the follow-up questionnaire at the 6- and 12-month follow-up and may also be recorded by facilitators through communication with participants in the intervention arm. We will also attempt to capture any unforeseen consequences in the qualitative interviews.

#### Expected events

Expected AEs include planned/elective hospitalisations, or unplanned but expected hospitalisation due to flare-up of IBD: these are expected during the course of the trial and will not be collected or reported as serious AEs (SAEs).

After a SAE, a decision will be made by the trial team, after advice from the relevant authorities and the participant’s IBD team, as to whether the participant should be withdrawn from either their randomised treatment or from the trial. However, we do not envisage a situation, except death, in which a participant would need to be withdrawn. Arrangements will be made by the trial team for further assessment and management as agreed with the relevant authorities, GP and participant.

The investigator will provide the trial team with a 1-month follow-up report on all SAEs. Further monthly reports should be provided in the absence of resolution. These reports will be communicated to the Programme Steering Committee, REC, and to the local R&D office. Blank adverse event forms will be distributed to sites that are recruiting.

## Ethics and dissemination

### Research ethics approval

The Chief Investigator has obtained approval from a recognised NRES Research Ethics Committee & Health Research Authority (HRA) (Additional file 2). The study will be conducted in accordance with the recommendations for physicians involved in research on human subjects adopted by the 18th World Medical Assembly, Helsinki 1964 (incl. later revisions) and any other relevant ethical guidance.

### Protocol amendments

After obtaining a favourable ethical opinion and HRA approval, any subsequent changes to the study conduct, design or management will be notified to the original approving REC & HRA and any other relevant regulatory authority via the UK Amendment process (http://www.hra.nhs.uk/research-community/during-your-research-project/amendments/).

### Consent

Written information about the RCT and an invite email/letter will be emailed or posted to potential participants who have previously completed the IBD-BOOST Survey (stage 2 of the research programme) and have consented to further approaches to participate in research. Participants will be able to participate by providing consent online on the study website or by returning a completed consent form by post (Additional file 4). There will be two reminders by text or email after 1 and 3 weeks for non-responders.

All participants are free to withdraw from the study at any time without giving reasons and without prejudicing further treatment. In line with GDPR guidelines, participant rights to access, change or remove their information will be limited. If a participant chooses to withdraw from the study, we will keep the information that we have already obtained. To safeguard participant rights, we will use the minimum personally identifiable information possible at all stages of the study. Study participants will notify the chief investigator and/or lead research team based at KCL if they wish to withdraw, using the contact details provided in patient information leaflets for the study and on the website.

## Confidentiality of participants

The Chief Investigator will preserve the confidentiality of participants taking part in the study and will work in accordance with the Caldicott Principles, Data Protection Act 2018, NHS Code of Confidentiality and any relevant NHS Trust organisational policies or other applicable Data Protection legislation. Data collected may be used to support other research in the future and may be shared anonymously with other researchers as stated in the PIL and consent form subject to the necessary regulatory approvals being in place.

## Access to data

Trial investigators and the relevant members of the study team (i.e. trial statistician, health economist and those involved in process evaluation) at KCL and the PCTU will have access to the final trial data set. The sponsor will archive trial data including identifiable information for 10 years after the trial has finished.

## Ancillary and post-trial care

The study is sponsored by the LNWUH NHS Trust; the NHS Litigation Authority (NHSLA) Indemnity scheme will cover the study.

## Dissemination

For authorship eligibility, we will follow the recognised guidelines for authorship (https://www.bmj.com/about-bmj/resources-authors/article-submission/authorship-contributorship).

It is not intended to use professional writers.

Participants can request copies of a lay summary of the final report of the outcome of the study by indicating that they want this and giving permission to store contact details for this purpose on the consent form.

### To health professionals who could develop services, and the academic community

We will submit results for publication in multidisciplinary academic journals (such as Inflammatory Bowel Diseases and Journal of Crohn’s & Colitis) to disseminate to professional audiences. We will submit to key IBD conferences, including the UK British Society of Gastroenterology, the European Crohn’s & Colitis Organisation and the USA Digestive Diseases Week.

Anticipated professional publications include:

- Primary and secondary outcomes paper
- Health Economics evaluation paper
- Mediators and moderators of outcome
- Process Evaluation paper
- Study Protocol (current paper)

We will also work with our patient and public volunteers, training those who are willing to present results at local and regional Crohn’s & Colitis UK meetings. We will alongside patients construct a user-friendly lay summary for the CCUK newsletter and website. We will prepare a more detailed summary of results in lay language for participants and people with IBD who request this and adapt the charity information sheet on bowel control, fatigue and pain accordingly. We will discuss dissemination via their newsletter with the European Federation of Crohn’s & Colitis (patient) Associations. The study team are members of all these groups.

## Patient and public involvement (PPI)

People with IBD have been extensively involved in developing this research. In particular, PPI has informed or will inform:

- Identification of the research questions for the programme
- Development of the intervention
- Design of the research (including development of patient-facing materials)
- Management of the research
- Undertaking the research
- Analysis of results
- Dissemination of findings

## Discussion

This will be the first large-scale RCT of symptom management in IBD and the first to attempt to address multiple symptoms in the same intervention. The intervention has been rigorously developed using a theoretically based logic model. If the online self-management programme for those with IBD-related symptoms of fatigue, pain and urgency is shown to improve patients’ quality of life, this trial will enable clinicians and patients to make informed management decisions. The intervention has been designed to be easily scalable and implemented. This is to our knowledge the first trial focused on multiple symptoms that have been prioritised by both people with IBD and health professionals. If the intervention improves Quality of Life, it has potential to be adapted to the same symptoms of fatigue, pain and urgency in other conditions or for additional symptoms of IBD to be added.

## Trial status

Protocol version 3.0 (16.06.2020). Ethics approval has been obtained and site set-up is in progress. We commenced recruitment in January 2020 and anticipate completing recruitment of 680 participants in July 2022.

## Supplementary Information

**Additional file 1.** SPIRIT checklist.

**Additional file 2.** Ethics approval document.

**Additional file 3.** Original funding document.

**Additional file 4.** Consent form.

**Additional file 5.** Management of biological specimens (faecal calprotectin).

## Data Availability

Not applicable.

## Abbreviations

AE: Adverse event
CCUK: Crohn’s & Colitis UK (registered charity supporting people with IBD)
CD: Crohn’s disease
CI: Chief Investigator
CRF: Case report form
FI: Faecal incontinence
GDPR: General Data Protection Regulation
HRA: Health Research Authority
IBD: Inflammatory bowel disease
IBS: Irritable bowel syndrome
ISF: Investigator Site File
KCL: King’s College London
LNWH: London North West Healthcare NHS Trust
NHS: National Health Service
NICE: National Institute for Clinical Excellence
NIHR: National Institute for Health Research
NRES: National Research Ethics Service
PCTU: Pragmatic Clinical Trials Unit (Queen Mary University of London)
PFfAR: Programme Grant for Applied Research
PI: Principal Investigator (local collaborator / lead at each research site)
PMG: Programme Management Group
PPI: Patient and Public Involvement
PSC: Programme Steering Committee
QMUL: Queen Mary University of London
RCT: Randomised controlled trial
REC: Research Ethics Committee
SAE: Serious adverse event
TMF: Trial Master File
UC: Ulcerative Colitis
UK-IBDQ: UK version of the Inflammatory Bowel Disease Questionnaire

## References

[1] Wilson BS, Lonnfors S, Vermeire S, Greco M, Hommes DW, Bell C (2012). The true impact of IBD: a European Crohn’s and Ulcerative Colitis patient life impact survey 2010-11.

[2] Czuber-Dochan W, Dibley LB, Terry H, Ream E, Norton C (2013). The experience of fatigue in people with inflammatory bowel disease: an exploratory study. J Adv Nurs.

[3] Norton C, Dibley LB, Bassett P (2013). Faecal incontinence in inflammatory bowel disease: associations and effect on quality of life. J Crohn’s Colitis.

[4] Czuber-Dochan W, Norton C, Bredin F, Darvell M, Nathan I, Terry H (2014). Healthcare professionals’ perceptions of fatigue experienced by people with IBD. J Crohn’s Colitis.

[5] Dibley L, Norton C (2013). Experiences of fecal incontinence in people with inflammatory bowel disease: self-reported experiences among a community sample. Inflamm Bowel Dis.

[6] Lind AJ (2015). Top ten research priorities: inflammatory bowel disease.

[7] Donovan KA, Small BJ, Andrykowski MA, Munster P, Jacobsen PB (2007). Utility of a cognitive-behavioral model to predict fatigue following breast cancer treatment. Health Psychol.

[8] Harrison AM, McCracken LM, Bogosian A, Moss-Morris R (2015). Towards a better understanding of MS pain: a systematic review of potentially modifiable psychosocial factors. J Psychosom Res.

[9] Keefe FJ, Van Horn Y (1993). Cognitive-behavioral treatment of rheumatoid arthritis pain maintaining treatment gains. Arthritis Rheumatol.

[10] Van Kessel K, Moss-Morris R (2006). Understanding multiple sclerosis fatigue: a synthesis of biological and psychological factors. J Psychosom Res.

[11] Everitt HA, Landau S, O'Reilly G, Sibelli A, Hughes L, Windgassen S, et al. Cognitive behavioural therapy for irritable bowel syndrome: 24-month follow-up of participants in the ACTIB randomised trial. Lancet. 2019. 10.1016/S2468-1253(19)30243-2. 10.1016/S2468-1253(19)30243-2 PMC7026694 31492643

[12] Everitt HA, Landau S, O'Reilly G, Sibelli A, Hughes S, Windgassen S, et al. Assessing telephone-delivered cognitive-behavioural therapy (CBT) and web-delivered CBT versus treatment as usual in irritable bowel syndrome (ACTIB): a multicentre randomised trial. Gut. 2019. 10.1136/gutjnl-2018-317805. 10.1136/gutjnl-2018-317805 PMC6709776 30971419

[13] Moss-Morris R, Harrison AM, Safari R, Norton S, van der Linden ML, Picariello F, Thomas S, White C, Mercer T (2019). Which behavioural and exercise interventions targeting fatigue show the most promise in multiple sclerosis? A systematic review with narrative synthesis and meta-analysis. Behav Res Ther.

[14] Ahl A, Mikocka-Walus A, Gordon A, Andrews JM (2013). Are self-administered or minimal therapist contact psychotherapies an effective treatment for irritable bowel syndrome (IBS): a systematic review. J Psychosom Res.

[15] Eccleston C, Fisher E, Craig L, Duggan GB, Rosser BA, Keogh E. Psychological therapies (Internet-delivered) for the management of chronic pain in adults. Cochrane Database Syst Rev. 2014;2014(2):CD010152. 10.1002/14651858.CD010152.pub2. 10.1002/14651858.CD010152.pub2 PMC6685592 24574082

[16] Fisher E, Law E, Palermo TM, Eccleston C. Psychological therapies (remotely delivered) for the management of chronic and recurrent pain in children and adolescents. Cochrane Database Syst Rev. 2014. 10.1002/14651858.CD011118. 10.1002/14651858.CD011118.pub2 PMC4833498 25803793

[17] Moss-Morris R, McCrone P, Yardley L, van Kessel K, Wills G, Dennison L (2012). A pilot randomised controlled trial of an Internet-based cognitive behavioural therapy self-management programme (MS Invigor8) for multiple sclerosis fatigue. Behav Res Ther.

[18] Pajak R, Lackner J, Kamboj SK (2013). A systematic review of minimal-contact psychological treatments for symptom management in irritable bowel syndrome. J Psychosom Res.

[19] Lackner JM, Jaccard J, Keefer L, Brenner DM, Firth RS, Gudleski GD, Hamilton FA, Katz LA, Krasner SS, Ma CX, Radziwon CD, Sitrin MD (2018). Improvement in gastrointestinal symptoms after cognitive behavior therapy for refractory irritable bowel syndrome. Gastroenterolgy.

[20] Craig P, Dieppe P, Macintyre S, Michie S, Nazareth I, Petticrew M. Developing and evaluating complex interventions: the new Medical Research Council guidance. Br Med J. 2008. 10.1136/bmj.a1655. 10.1136/bmj.a1655 PMC2769032 18824488

[21] Norton C, Czuber-Dochan W, Artom M, Sweeney L, Hart A (2017). Systematic review: interventions for abdominal pain management in inflammatory bowel disease. Aliment Pharmacol Ther.

[22] Sweeney L, Moss-Morris R, Czuber-Dochan W, Murrells T, Norton C (2020). Developing a better biopsychosocial understanding of pain in inflammatory bowel disease: a cross-sectional study. Eur J Gastroenterol Hepatol.

[23] Artom M, Czuber-Dochan W, Sturt J, Norton C (2016). Targets for health interventions for inflammatory bowel disease-fatigue. J Crohn’s Colitis.

[24] Moss-Morris R, McAlpine L, Didsbury L, Spence M (2010). A randomized controlled trial of a cognitive behavioural therapy-based self-management intervention for irritable bowel syndrome in primary care. Psychol Med.

[25] Norton C (2004). Nurses, bowel continence, stigma, and taboos. J Wound Ostomy Continence Nurs.

[26] Norton C, Chelvanayagam S (2001). Methodology of biofeedback for adults with fecal incontinence: a program of care. J Wound Ostomy Continence Nurs.

[27] Norton C, Chelvanayagam S (2004). Bowel continence nursing.

[28] Harrison A, Silber E, McCracken L, Moss-Morris R (2015). Beyond a physical symptom: the importance of psychosocial factors in multiple sclerosis pain. Eur J Neurol.

[29] Everitt H, Moss-Morris R, Sibelli A, Tapp L, Coleman N, Yardley L, Smith P, Little P (2013). Management of irritable bowel syndrome in primary care: the results of an exploratory randomised controlled trial of mebeverine, methylcellulose, placebo and a self-management website. BMC Gastroenterol.

[30] Norton C, Chelvanayagam S, Wilson-Barnett J, Redfern S, Kamm MA (2003). Randomized controlled trial of biofeedback for fecal incontinence. Gastroenterology.

[31] Norton C, Dibley LB, Hart A, Duncan J, Emmanuel A, Knowles CH, Stevens N, Terry H, Verjee A, Kerry S, Hounsome N (2015). Faecal incontinence intervention study (FINS): self-management booklet information with or without nurse support to improve continence in people with inflammatory bowel disease: study protocol for a randomized controlled trial. Trials.

[32] Cross RK, Watson AR. Telemanagement of inflammatory bowel disease: New York: Springer; 2015.

[33] Elkjaer M, Shuhaibar M, Burisch J, Bailey Y, Scherfig H, Laugesen B, Avnstrøm S, Langholz E, O'Morain C, Lynge E, Munkholm P (2010). E-health empowers patients with ulcerative colitis: a randomised controlled trial of the web-guided ‘Constant-care’approach. Gut.

[34] Protheroe J, Rogers A, Kennedy AP, Macdonald W, Lee V (2008). Promoting patient engagement with self-management support information: a qualitative meta-synthesis of processes influencing uptake. Implement Sci.

[35] Kennedy AP, Rogers AE (2002). Improving patient involvement in chronic disease management: the views of patients, GPs and specialists on a guidebook for ulcerative colitis. Patient Educ Couns.

[36] McCombie A, Gearry R, Andrews J, Mulder R, Mikocka-Walus A (2015). Does computerized cognitive behavioral therapy help people with inflammatory bowel disease? A randomized controlled trial. Inflamm Bowel Dis.

[37] Lorig KR, Sobel DS, Stewart AL, Brown BW, Bandura A, Ritter P, Gonzalez VM, Laurent DD, Holman HR (1999). Evidence suggesting that a chronic disease self-management program can improve health status while reducing hospitalization: a randomized trial. Med Care.

[38] Michie S, Richardson M, Johnston M, Abraham C, Francis J, Hardeman W, Eccles MP, Cane J, Wood CE (2013). The behavior change technique taxonomy (v1) of 93 hierarchically clustered techniques: building an international consensus for the reporting of behavior change interventions. Ann Behav Med.

[39] Beck JS. Cognitive behavior therapy: basics and beyond: New York: Guilford Press; 2011.

[40] Bodger K, Ormerod C, Shackcloth D, Harrison M. Development and validation of a rapid, generic measure of disease control from the patient’s perspective: the IBD-control questionnaire. Gut. 2014;63(7):1092–102. 10.1136/gutjnl-2013-305600. 10.1136/gutjnl-2013-305600 PMC4078750 24107590

[41] Artom M, Czuber-Dochan W, Sturt J, Norton C (2017). Cognitive behavioural therapy for the management of inflammatory bowel disease-fatigue with a nested qualitative element: study protocol for a randomised controlled trial. Trials.

[42] Deale A, Chalder T, Mark SI, Wessely S (1997). Cognitive behavior therapy for chronic fatigue syndrome: a randomized controlled trial. Am J Psychiatry.

[43] van Kessel K, Moss-Morris R, Willoughby E, Chalder T, Johnson MH, Robinson E (2008). Cognitive behavior therapy compared to relaxation training for multiple sclerosis fatigue: a randomized controlled trial. Psychosom Med.

[44] Moss-Morris R, Dennison L, Landau S, Yardley L, Silber E, Chalder T (2013). A randomized controlled trial of cognitive behavioral therapy (CBT) for adjusting to multiple sclerosis (the saMS trial): does CBT work and for whom does it work?. J Consult Clin Psychol.

[45] Cole F, Howden-Leach H, Macdonald H, Carus C (2012). Overcoming chronic pain: a self-help guide using cognitive behavioural techniques.

[46] Lewin R (2010). The pain management plan: how people living with pain found a better life. The things that helped them and the things that set them back.

[47] Cheung W-Y, Garratt AM, Russell IT, Williams JG (2000). The UK IBDQ—a British version of the inflammatory bowel disease questionnaire. J Clin Epidemiol.

[48] Vaizey C, Carapeti E, Cahill J, Kamm M (1999). Prospective comparison of faecal incontinence grading systems. Gut.

[49] Maeda Y, Parés D, Norton C, Vaizey CJ, Kamm MA (2008). Does the St. Mark’s incontinence score reflect patients’ perceptions? A review of 390 patients. Dis Colon Rectum.

[50] Czuber-Dochan W, Norton C, Bassett P, Berliner S, Bredin F, Darvell M, Forbes A, Gay M, Nathan I, Ream E, Terry H (2014). Development and psychometric testing of inflammatory bowel disease fatigue (IBD-F) patient self-assessment scale. J Crohn’s Colitis.

[51] Norton C, Czuber-Dochan W, Bassett P, Berliner S, Bredin F, Darvell M, Forbes A, Gay M, Ream E, Terry H (2015). Assessing fatigue in inflammatory bowel disease: comparison of three fatigue scales. Aliment Pharmacol Ther.

[52] Herdman M, Gudex C, Lloyd A, Janssen M, Kind P, Parkin D, Bonsel G, Badia X (2011). Development and preliminary testing of the new five-level version of EQ-5D (EQ-5D-5L). Qual Life Res.

[53] Kroenke K, Spitzer R, Williams J. The PHQ-9: validity of a brief depression severity measure. [Research support]. Non-US Gov’t. J Gen Intern Med. 2001;16(9):606–13. 10.1046/j.1525-1497.2001.016009606.x. 10.1046/j.1525-1497.2001.016009606.x PMC1495268 11556941

[54] Lorig KR, Sobel DS, Ritter PL, Laurent D, Hobbs M (2001). Effect of a self-management program on patients with chronic disease. Effect Clin Pract.

[55] Broadbent E, Petrie KJ, Main J, Weinman J (2006). The brief illness perception questionnaire. J Psychosom Res.

[56] Labus J, Bolus R, Chang L, Wiklund I, Naesdal J, Mayer E (2004). The Visceral Sensitivity Index: development and validation of a gastrointestinal symptom-specific anxiety scale. Aliment Pharmacol Ther.

[57] Kyaw MH, Moshkovska T, Mayberry J (2014). A prospective, randomized, controlled, exploratory study of comprehensive dietary advice in ulcerative colitis: impact on disease activity and quality of life. Eur J Gastroenterol Hepatol.

[58] Williams JG, Alam MF, Alrubaiy L, Clement C, Cohen D, Grey M (2016). Comparison Of iNfliximab and ciclosporin in STeroid Resistant Ulcerative Colitis: pragmatic randomised Trial and economic evaluation (CONSTRUCT). Health Technol Assess (Winchester, England).

[59] Kennedy A, Nelson E, Reeves D, Richardson G, Roberts C, Robinson A, Rogers AE, Sculpher M, Thompson DG (2004). A randomised controlled trial to assess the effectiveness and cost of a patient orientated self management approach to chronic inflammatory bowel disease. Gut.

[60] Drossman D, Thompson WG, Talley N, Funch-Jensen P, Janssens J, Whitehead W (1990). Identification of sub-groups of functional gastrointestinal disorders. Gastroenterol Int.

[61] Edwards P, Roberts I, Clarke M, DiGuiseppi C, Pratap S, Wentz R, Kwan I (2002). Increasing response rates to postal questionnaires: systematic review. BMJ.

[62] Excellence NIfHaC (2013). Guide to the methods of technology appraisal.

[63] Glick HA, Doshi JA, Sonnad SS, Polsky D (2014). Economic evaluation in clinical trials.

[64] Moore GF, Audrey S, Barker M, Bond L, Bonell C, Hardeman W (2015). Process evaluation of complex interventions: Medical Research Council guidance. BMJ.

[65] Ritchie J, Lewis J, Nicholls CM, Ormston R. Qualitative research practice: a guide for social science students and researchers. Thousand Oaks: Sage; 2013.

[66] Dibley L, Norton C, Cotterill N, Bassett P (2014). Development and initial validation of a new assessment tool for faecal incontinence in inflammatory bowel disease: the International Consultation on Incontinence Questionnaire–Inflammatory Bowel Disease (iciq-ibd). Colorectal Dis.

[67] Garfield S, Jheeta S, Husson F, Jacklin A, Bischler A, Norton C, Franklin BD (2016). Lay involvement in the analysis of qualitative data in health services research: a descriptive study. Res Involv Engagem.

[68] Garfield S, Jheeta S, Jacklin A, Bischler A, Norton C, Franklin BD (2015). Patient and public involvement in data collection for health services research: a descriptive study. Res Involv Engagem.

[69] Dibley L, Czuber-Dochan W, Woodward S, Bassett P, Sturt J, Bellamy A, et al., editors. Distress in inflammatory bowel disease: development of a new assessment tool. J Crohn’s Colitis. Oxford: Oxford Univ Press; 2016.

[70] De Vos M, Jahnsen J, Vandervoort J, D’haens G, Dewit O, Louis E, et al., editors. Use of fecal calprotectin as marker of disease activity in patients under maintenance treatment with infliximab for ulcerative colitis. 7th Congress of ECCO. Inflamm Bowel Dis. 2013;19(10):2111–7. 10.1097/MIB.0b013e31829b2a37. 10.1097/MIB.0b013e31829b2a37 23883959

