# Supplementary material for: Supported online self-management versus care as usual for symptoms of fatigue, pain and urgency/incontinence in adults with inflammatory bowel disease (IBD-BOOST): study protocol for a randomised controlled trial
Source: Trials. 2021 Aug 3;22:516. doi: 10.1186/s13063-021-05466-4 (PMC8329619; doi:10.1186/s13063-021-05466-4)
Supplement: Supplementary file 4 — Additional file 4. Consent form. [file 13063_2021_5466_MOESM4_ESM.pdf]

IBD-BOOST TRIAL						
SITE ID:						
PARTICIPANT ID:						
CRF BOOKLET NUMBER:						

CONSENT FORM (ONLINE)

Title: A Randomised Controlled Trial of supported online self-management for symptoms of fatigue, pain and urgency/incontinence in people with inflammatory bowel disease: the IBD-BOOST trial

Chief Investigator: Professor Christine Norton

Please read the following statements carefully and tick YES if you agree. We will not collect your information or ask you to participate in this trial without your consent.

	YES	NO
1. I confirm that I have read and understood the information sheet [Standard or shortened PIL to be referenced depending on randomisation Version: X.X XX/XX/XXXX] for the above trial. I have had the opportunity to consider the information, ask questions using the contact details on the information sheet and have had these answered satisfactorily.	☐	☐
2. I understand that my participation is voluntary (my choice) and that I am free to withdraw at any time without giving any reason, without my medical care or legal rights being affected.	☐	☐
3. I understand that relevant information from my medical records and data that I have provided as part of this trial may be accessed by individuals from King's College London, the sponsor, the Pragmatic Clinical Trials Unit at Queen Mary University of London, from relevant regulatory authorities or from the NHS trust, where it is relevant to my taking part in this research. Only information relevant and necessary for my participation in this research will be accessed. I give permission for these individuals to have access to my records.	☐	☐
4. I understand that my IBD healthcare team may be notified of my involvement in this trial.	☐	☐
5. I consent to completing a stool sample that will be processed at a Laboratory at King's College Hospital. I understand this will not be required if I participated in the IBD-BOOST Optimise study.	☐	☐
6. I understand that the information collected about me may be used to support other research in the future and may be shared anonymously with other researchers.	☐	☐

IBD-BOOST TRIAL						
SITE ID:						
PARTICIPANT ID:						
CRF BOOKLET NUMBER:						

	Yes	No
7. I agree to the identifiable data that I have completed for the previous IBD-BOOST Survey and, if applicable, the IBD-BOOST Optimise study, being used for this trial.	☐	☐

	YES	NO
8. Please tick yes if you agree to being in the trial.	☐	☐

‘By typing my first name and last name below, I give consent to participating in the IBD-BOOST TRIAL’.

First Name of Participant	Last Name of Participant	Date

As part of the trial, we would like to conduct interviews either by phone or face-to-face with some of the people taking part. This will help us understand more about their experience of symptoms and the self-management programme.

The following statement is optional. Please tick YES if you agree.

	YES	NO
I agree to the research team contacting me about taking part in interviews.	☐	☐

If you need more information to help you decide, please contact: [*insert name & role*]
Email: [*inset name*], **Tel:** [*insert number*]

FUNDED BY NIHR | National Institute for Health Research
