# Supplementary material for: Supported online self-management versus care as usual for symptoms of fatigue, pain and urgency/incontinence in adults with inflammatory bowel disease (IBD-BOOST): study protocol for a randomised controlled trial
Source: Trials. 2021 Aug 3;22:516. doi: 10.1186/s13063-021-05466-4 (PMC8329619; doi:10.1186/s13063-021-05466-4)
Supplement: Supplementary file 5 — Additional file 5. Management of biological specimens (faecal calprotectin). [file 13063_2021_5466_MOESM5_ESM.docx]

Additional file 5: Plans for collection, laboratory evaluation, and storage of biological specimens for genetic or molecular analysis in the current trial

We will collect faecal calprotectin results as a measure of inflammation for all participants who have not previously participated in the IBD-BOOST Optimise study (81). Calprotectin results will be obtained by posting sample kits to participants after the baseline questionnaire has been completed. There will be one reminder by email or text for non-responders. The kits will include a standard and widely used faecal sample pot; packaging and postage will be compliant with 2004 Human Tissue Act. The envelope will have pre-paid postage. The pot sample will be labelled with the participants study ID only. All samples will be processed by King’s College Hospital laboratory. No identifying information will be sent to the laboratory at King’s College Hospital. All samples will be disposed of following analysis. Results for calprotectin tests on stool samples provided by the participant will be accessed by the central research team on King’s College Hospital laboratory’s secure results portal and uploaded on to the study database on the Calprotectin CRF. A copy of the result will be emailed to the participant along with a brief explanation using a template, advice on further actions that should be taken and contact details for more advice.
