# Supplementary material for: Supported online self-management versus care as usual for symptoms of fatigue, pain and urgency/incontinence in adults with inflammatory bowel disease (IBD-BOOST): study protocol for a randomised controlled trial
Source: Trials. 2021 Aug 3;22:516. doi: 10.1186/s13063-021-05466-4 (PMC8329619; doi:10.1186/s13063-021-05466-4)
Supplement: Supplementary file 2 — Additional file 2. Ethics approval document. [file 13063_2021_5466_MOESM2_ESM.pdf]

Health Research Authority

London - Surrey Research Ethics Committee
Nottingham Centre
The Old Chapel
Royal Standard Place
Nottingham
NG1 6FS

Telephone: 0207 1048310

Please note: This is the favourable opinion of the REC only and does not allow you to start your study at NHS sites in England until you receive HRA Approval

12 July 2019

Professor Christine Norton
Florence Nightingale Professor of Clinical Nursing Research
King's College London
57 Waterloo Road
London
SE1 8WA

Dear Professor Norton[##IfStudent##]

**Study title:**	**A Randomised Controlled Trial of supported online self-management for symptoms of fatigue, pain and urgency/incontinence in people with inflammatory bowel disease: the IBD-BOOST trial**
**REC reference:**	**19/LO/0750**
**Protocol number:**	**1.0[##IfProtocolRef##]**
**IRAS project ID:**	**258725**

Thank you for your letter of 20 June 2019, responding to the Committee's request for further information on the above research [and submitting revised documentation].

The further information has been considered on behalf of the Committee by the Chair together with Miss Malins.

Confirmation of ethical opinion

On behalf of the Committee, I am pleased to confirm a favourable ethical opinion for the above research on the basis described in the application form, protocol and supporting documentation [as revised], subject to the conditions specified below.

Conditions of the favourable opinion

The REC favourable opinion is subject to the following conditions being met prior to the start of the study.

Confirmation of Capacity and Capability (in England, Northern Ireland and Wales) or NHS management permission (in Scotland) should be sought from all NHS organisations involved in the study in accordance with NHS research governance arrangements. Each NHS organisation must confirm through the signing of agreements and/or other documents that it has given permission for the research to proceed (except where explicitly specified otherwise).

Guidance on applying for HRA and HCRW Approval (England and Wales)/ NHS permission for research is available in the Integrated Research Application System.

For non-NHS sites, site management permission should be obtained in accordance with the procedures of the relevant host organisation.

Sponsors are not required to notify the Committee of management permissions from host organisations

Registration of Clinical Trials

It is a condition of the REC favourable opinion that all clinical trials are registered on a publicly accessible database. For this purpose, clinical trials are defined as the first four project categories in IRAS project filter question 2. For clinical trials of investigational medicinal products (CTIMPs), other than adult phase I trials, registration is a legal requirement.

Registration should take place as early as possible and within six weeks of recruiting the first research participant at the latest. Failure to register is a breach of these approval conditions, unless a deferral has been agreed by or on behalf of the Research Ethics Committee (see here for more information on requesting a deferral: https://www.hra.nhs.uk/planning-and-improving-research/research-planning/research-registration-research-project-identifiers/

As set out in the UK Policy Framework, research sponsors are responsible for making information about research publicly available before it starts e.g. by registering the research project on a publicly accessible register. Further guidance on registration is available at: https://www.hra.nhs.uk/planning-and-improving-research/research-planning/transparency-responsibilities/

You should notify the REC of the registration details. We will audit these as part of the annual progress reporting process.

It is the responsibility of the sponsor to ensure that all the conditions are complied with before the start of the study or its initiation at a particular site (as applicable).

After ethical review: Reporting requirements

The attached document “After ethical review – guidance for researchers” gives detailed guidance on reporting requirements for studies with a favourable opinion, including:

- Notifying substantial amendments
- Adding new sites and investigators
- Notification of serious breaches of the protocol
- Progress and safety reports
- Notifying the end of the study, including early termination of the study
- Final report

The latest guidance on these topics can be found at https://www.hra.nhs.uk/approvals-amendments/managing-your-approval/.

Ethical review of research sites

NHS/HSC sites

The favourable opinion applies to all NHS/HSC sites listed in the application subject to confirmation of Capacity and Capability (in England, Northern Ireland and Wales) or management permission (in Scotland) being obtained from the NHS/HSC R&D office prior to the start of the study (see "Conditions of the favourable opinion" below).

Non-NHS/HSC sites

I am pleased to confirm that the favourable opinion applies to any non-NHS/HSC sites listed in the application, subject to site management permission being obtained prior to the start of the study at the site.

Approved documents

The final list of documents reviewed and approved by the Committee is as follows:

Document	*Version*	*Date*
Interview schedules or topic guides for participants [Appendix 25; IBD-BOOST TRIAL; Topic guides for all process evaluation interviews]	1.0	14 March 2019
IRAS Application Form [IRAS_Form_20062019]		20 June 2019
IRAS Application Form XML file [IRAS_Form_20062019]		20 June 2019
IRAS Checklist XML [Checklist_21062019]		21 June 2019
Letter from funder [Funding award letter RP-PG-0216-20001 Notification Letter]		30 March 2016
Other [Appendix 3; Protocol for a Study within a Trial]	1.0	04 April 2019
Other [Appendix 4; IBD-BOOST TRIAL General Health Assessment]	1.0	04 April 2019
Other [Appendix 10; IBD-BOOST TRIAL General health Assessment Eligibility criteria and email template]	1.0	04 April 2019

Other [Appendix 13; IBD-BOOST TRIAL Logic Model for Intervention]	1.0	14 March 2019
Other [Appendix 15; IBD-BOOST TRIAL Template email & text to return outcome measures]	1.0	14 March 2019
Other [Appendix 16; IBD-BOOST TRIAL Reminder to return calprotectin sample (email or text)]	1.0	14 March 2019
Other [Appendix 18; IBD-BOOST TRIAL Email Informing participants of calprotectin result]	1.0	14 March 2019
Other [Appendix 19; IBD-BOOST TRIAL 6 and 12 month letter accompanying follow-up incentive]	1.0	14 March 2019
Other [Appendix 20; IBD-BOOST TRIAL Email informing participants of randomisation allocation]	1.0	14 March 2019
Other [Appendix 21; IBD-BOOST TRIAL CRF for recording unplanned facilitator time spent on the intervention]	1.0	04 April 2019
Other [Appendix 22; IBD-BOOST TRIAL Website Analytics CRF]	1.0	04 April 2019
Other [Appendix 27; IBD-BOOST TRIAL; Consent forms (clinicians) for process evaluation interviews]	1.0	14 March 2019
Other [Appendix 28; IBD-BOOST TRIAL GANTT chart]	1.0	14 March 2019
Other [Appendix 29. IBD-BOOST TRIAL PHQ9 risk assessment]	1.0	04 April 2019
Other [Appendix 9; IBD-BOOST TRIAL Reminder Email and text template]	1.0	14 March 2019
Other [Appendix 12; IBD-BOOST TRIAL 6 and 12 Month Follow Up questionnaire CRF]	1.0	04 April 2019
Other [Appendix 14; IBD-BOOST TRIAL Sample text for online session one]	1.0	14 March 2019
Other [Appendix 17; IBD-BOOST TRIAL CRF for reporting results of faecal calprotectin test]	1.0	14 March 2019
Other [Appendix 7; IBD-BOOST TRIAL Email Invite Template Version 2.0 07.06.2019]	2.0	07 June 2019
Other [Appendix 26; IBD-BOOST TRIAL; Process Evaluation Consent forms (patient participants) Version 2.0 07.06.2019]	2.0	07 June 2019
Other [Appendix 6; IBD-BOOST TRIAL PiL (Shortened)]	2.0	07 June 2019
Other [Appendix 23; IBD-BOOST TRIAL; Process evaluation interviews PIS (patients) Version 2.0 07.06.2019]	2.0	07 June 2019
Other [Appendix 24; IBD-BOOST TRIAL; Process Evaluation Interviews PIS (staff) Version 2.0 07.06.19]	2.0	07 June 2019
Participant consent form [Appendix 8; IBD-BOOST TRIAL Consent Form]	2.0	07 June 2019
Participant information sheet (PIS) [Appendix 5; IBD-BOOST TRIAL PiL (Standard)]	2.0	07 June 2019
Referee's report or other scientific critique report [Funder's final review]	8.0	17 March 2011
Research protocol or project proposal [IBD-BOOST TRIAL Protocol]	1.0	01 April 2019
Summary CV for Chief Investigator (CI) [CV for CI]		03 January 2019
Validated questionnaire [Appendix 11; IBD-BOOST TRIAL Baseline questionnaire CRF]		

Statement of compliance

The Committee is constituted in accordance with the Governance Arrangements for Research

Ethics Committees and complies fully with the Standard Operating Procedures for Research Ethics Committees in the UK.

User Feedback

The Health Research Authority is continually striving to provide a high quality service to all applicants and sponsors. You are invited to give your view of the service you have received and the application procedure. If you wish to make your views known please use the feedback form available on the HRA website:
http://www.hra.nhs.uk/about-the-hra/governance/quality-assurance/

HRA Learning

We are pleased to welcome researchers and research staff to our HRA Learning Events and online learning opportunities– see details at:
https://www.hra.nhs.uk/planning-and-improving-research/learning/

**19/LO/0750**	**Please quote this number on all correspondence**

With the Committee's best wishes for the success of this project.

Yours sincerely

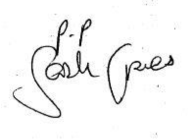

Mrs Chrissie Lawson
Chair

Email:nrescommittee.secoast-surrey@nhs.net

Enclosures: "After ethical review – guidance for researchers" *[SL-AR2]*

Copy to: Mr Simon Lewis, London North West University Healthcare NHS Trust
