# Supplementary material for: Supported online self-management versus care as usual for symptoms of fatigue, pain and urgency/incontinence in adults with inflammatory bowel disease (IBD-BOOST): study protocol for a randomised controlled trial
Source: Trials. 2021 Aug 3;22:516. doi: 10.1186/s13063-021-05466-4 (PMC8329619; doi:10.1186/s13063-021-05466-4)
Supplement: Supplementary file 3 — Additional file 3. Original funding document. [file 13063_2021_5466_MOESM3_ESM.pdf]

National Institute for Health Research

Professor Christine Norton
King's College London
Faculty of Nursing & Midwifery
57 Waterloo Road
London
United Kingdom
SE1 8WA

Central Commissioning Facility
Grange House
15 Church Street
Twickenham
TW1 3NL

Tel: 020 8843 8000
Fax: 020 8843 8001
Email: info@nihr-ccf.org.uk
Web: www.ccf.nihr.ac.uk

30 December 2016

Dear Professor Norton,

Programme Grants for Applied Research: RP-PG-0216-20001
Application Title: Living well with inflammatory bowel disease: optimising management of systems of fatigue, abdominal pain and faecal urgency/incontinence via tailored online self-mangement: the IBD-BOOST programme

Thank you for your application to the Programme Grants for Applied Research funding scheme. Your proposal has been reviewed by the Programme Grants for Applied Research Main Selection Panel, and I am now in a position to provide you with formal notification of the outcome of this assessment process.

As indicated in the Stage 2 application guidance, the selection criteria were as follows:

- The strength of the research team, including the relevant expertise and track-record of the applicants in conducting high-quality applied health research
- The relevance of the proposed research to the priorities and needs of the NHS
- The likelihood of significant benefit to the NHS and patients within three to five years of the end of the funding period
- The quality of the research planned
- The value for money provided by the proposal

Three expert sub-panels were convened to assist the Programme Grants for Applied Research Main Selection Panel. All sub-panels reviewed the Stage 2 applications against the above criteria. Their decision-making was also influenced by comments provided by peer reviewers and applicants' responses to these. Any unduly harsh remarks, bias or misunderstanding in these comments will have been taken into consideration by the sub-panels.

The sub-panels' recommendations were then reviewed by the Main Selection Panel, which made final funding recommendations across all topic areas. These funding recommendations were then considered, and approved, by the Director of Research and Development at the Department of Health.

I am pleased to be able to inform you that it is our intention to award you a Programme Grant. The sub-panel did, however, identify a number of concerns which would need to be satisfactorily addressed before funding could be awarded. The specific feedback of the sub-panel is being prepared and will be forwarded to you together with feedback about application finances and intellectual property.

In the first instance, please confirm your intention to take up this offer of funding by writing to programme.grants@nihr.ac.uk no later than **6 January 2017**.

We must ask you to refrain from publicising this funding recommendation until negotiations have been completed and a contract has been signed.

The call for proposals for the 23rd funding round will be open 1 Febuary 2017, details of which are currently available on our website (http://www.nihr.ac.uk/funding-and-support/funding-for-research-studies/how-to-apply/research-programmes/programme-grants-for-applied-research/pgfar-current-and-future-calls.htm).

Yours sincerely,

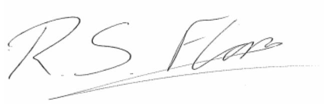

Rajinder Flora
Assistant Director
Programme Grants for Applied Research Programme
